# The use of artificial intelligence for delivery of essential health services across WHO regions: a scoping review

**DOI:** 10.3389/fpubh.2023.1102185

**Published:** 2023-07-04

**Authors:** Joseph Chukwudi Okeibunor, Anelisa Jaca, Chinwe Juliana Iwu-Jaja, Ngozi Idemili-Aronu, Housseynou Ba, Zukiswa Pamela Zantsi, Asiphe Mavis Ndlambe, Edison Mavundza, Derrick Muneene, Charles Shey Wiysonge, Lindiwe Makubalo

**Affiliations:** ^1^World Health Organization Regional Office for Africa, Brazzaville, Republic of Congo; ^2^Cochrane South Africa, South African Medical Research Council, Cape Town, South Africa; ^3^Department of Sociology/Anthropology, University of Nigeria, Nsukka, Nigeria; ^4^World Health Organization, Geneva, Switzerland; ^5^HIV and Other Infectious Diseases Research Unit, South African Medical Research Council, Durban, South Africa

**Keywords:** artificial intelligence, deep learning, machine learning, non-communicable diseases, communicable diseases artificial intelligence, communicable diseases

## Abstract

**Background:**

Artificial intelligence (AI) is a broad outlet of computer science aimed at constructing machines capable of simulating and performing tasks usually done by human beings. The aim of this scoping review is to map existing evidence on the use of AI in the delivery of medical care.

**Methods:**

We searched PubMed and Scopus in March 2022, screened identified records for eligibility, assessed full texts of potentially eligible publications, and extracted data from included studies in duplicate, resolving differences through discussion, arbitration, and consensus. We then conducted a narrative synthesis of extracted data.

**Results:**

Several AI methods have been used to detect, diagnose, classify, manage, treat, and monitor the prognosis of various health issues. These AI models have been used in various health conditions, including communicable diseases, non-communicable diseases, and mental health.

**Conclusions:**

Presently available evidence shows that AI models, predominantly deep learning, and machine learning, can significantly advance medical care delivery regarding the detection, diagnosis, management, and monitoring the prognosis of different illnesses.

## 1. Introduction

Artificial intelligence (AI) refers to the simulation of intellectual human behavior by computers. AI can be designed using lots of algorithms including machine learning (ML), deep learning (DL), natural language processing (NLP), support vector machine (SVM), and the artificial neural network (ANN) (1). These algorithms assist the system to identify the expected response which informs the computer what to expect (2). ML is the technique used in precision medicine for predicting treatment procedures and disease outcomes in patients (3). On the other hand, DL is a form of AI technique that is used health care to identify potential cancerous cells using in radiology images beyond what can be perceived by the human eye. This method can promote faster learning without being prompted (3). Another form of AI, i.e., NLP is related to the use of software programming to understand and manipulate natural language text or speech for practical purposes (4). This involves dealing with large volumes of clinical data and health literacy in the health sector (5). SVM is an algorithm used to assemble a classification system for model classification and trend. The ANN model is used to comprehend the reasoning and functioning of connection between neurons (1). ANN has been used to solve different issues by building mathematical models that imitate natural activities of the brain (1).

AI has several advantages, i.e., it is reliable, cost-effective, solves complex issues, and limits data loss (6). AI is applied in fields including business, engineering, or medical care. In medical care, this technology is used for diagnosis, therapy, and prognosis (7). AI is a rapidly evolving field in medical care, with great potential to inform evidence-based decision making and ultimately improve health outcomes. It has been applied across various fields including robotics, medical diagnosis, medical statistics, and human biology (8). This technology plays a role in addressing certain issues within the health system which comprise staff shortages, poor administration of health services (e.g., billing, repayments, and insurance fraud exposure), and poor infrastructure; to support the delivery of high-quality healthcare (4, 7). AI also has the potential to impact on several aspects, including clinical decision at points of care, drug research, and disease predictions, amongst others. This has been said to improve efficiency, safety, and access to medical care services (2, 6, 8, 9).

Therefore, the AI technology is necessary to help manage of medical care services, to make decisions concerning disease prediction, diagnosing and treatment plans for patients (10). The current challenges (e.g., difficulty accessing health facilities in time, poor quality of health care, staff shortages) within the health system of low- and middle-income countries (LMICs) warrant the implementation and use of this technology (10). It is likely that this technology is predominantly applied in high-income countries (HICs) as LMICs may not have the infrastructure for the technology in their healthcare systems (10). AI can be used to manage various diseases, namely, diabetes, cancer, emerging infectious diseases, sexually transmitted diseases, and mental health illnesses. This technique has been utilized to predict risk and diagnosis of diabetes predicated on genomic and EHR data, respectively (11). It has also been used to predict risk of complications such as nephropathy and retinopathy (11). In cancer, AI can be used to analyze imaging data obtained during routine cancer care, i.e., disease classification, detection, segmentation, characterization, and monitoring. This saves time and helps radiologists achieve better outcomes and identify cancerous lesions that could be missed by humans (12). Furthermore, AI models are also useful in predicting the progression of disease and mortality in patients infected with emerging infectious diseases, namely, the severe acute respiratory syndrome (SARS), H1N1 influenza virus, Middle East respiratory syndrome coronavirus (MERS-CoV) as it has been recently done in SARS-CoV2 (13). Additionally, this method has been widely applied and has been used as an intervention for mental health issues. AI has been reported to be effective in managing mental health issues, i.e., reducing anxiety through detecting emotional changes and thought patterns and increasing thinking styles (14).

AI applications in mental health can bring insights into new treatment approaches. This technique has also been used to predict the diagnosis of sexually transmitted infections including HIV, as these are global public health concerns (15). This method has also been used in HIV prevention such as identifying potential PrEP candidates at risk of infection in Kenya and Uganda (16). One study evaluated the performance of AI in predicting HIV, syphilis, gonorrhea, chlamydia in an Australian cohort among men who have sex with men and reported that this technique is accurate (17). This type of research has mainly been conducted in Belgium, China, Italy, Korea, Turkey, and USA while there needs to be research conducted in LMICs, specifically in Africa (11, 13–16).

While health professionals in HICs may have the expertise to use the AI techniques, there may be a serious need to build capacity around this technology among professionals in LMICs (10). This implies that this technology would not work in LMICs where health care practitioners do not have the capacity to apply AI and interpret AI results. It is, however, necessary to assess where and for what conditions AI is being used around the world, thus the need for this scoping review. The objective of the review was to map out and synthesize the available evidence on the use of AI to deliver medical care services, globally and regionally.

## 2. Materials and methods

We conducted a scoping review as per the methodology defined by Arskey and O'Malley (18). A scoping review is a methodology that is used to chart key concepts and evidence available in a particular field. The field of AI is rapidly developing hence we used this methodology to undertake this review.

### 2.1. Search strategy

Two authors (Anelisa Jaca and Chinwe Juliana Iwu-Jaja) conducted a search in PubMed on 07 March 2022 and Scopus on 16 March 2022. The following combination of key words was used for the search: (“Artificial intelligence” OR AI OR “machine learning” OR “machine intelligence” OR “deep learning”) AND (“health care” OR health OR “health delivery”). No language or date restrictions were employed. We first developed and implemented a search strategy in PubMed, which was afterwards adapted for Scopus.

### 2.2. Study selection

Titles and abstracts of identified records were independently screened by two researchers, Zukiswa Pamela Zantsi (ZPZ) and Asiphe Mavis Ndlambe (AMN), to identify potentially eligible records. Abstracts of records judged to be potentially eligible by one or both researchers were re-screened by a second pair of more experienced researchers, Anelisa Jaca (AJ) and Chinwe Juliana Iwu-Jaja (CJI). The latter made the decision on potentially eligible studies through discussion and consensus. AJ and CJI then assessed the full text of potentially eligible studies and included publications of primary studies which: reported on the use of AI, addressed a health condition, assessed the effectiveness of the AI method used, and were published in a peer-reviewed journal in English. We excluded reviews.

### 2.3. Data extraction and analysis

A piloted data extraction form containing a list of data of interest and their definitions was used to extract data from eligible studies. Data were extracted independently by AJ, ZPZ, CJI, AMN, and Edison Mavundza (EM). The extracted data included the first author's name, year of publication, study population, country where the study was conducted, aim of the study, health issue, AI method, application of AI, and findings. The WHO region and category of health issue were also charted. We used a narrative synthesis method to analyse and report the key concepts and findings related to AI applications on medical care delivery. We did not evaluate the methodological quality of included studies since our aim was to identify and map the available evidence on the use of AI to deliver essential medical services. Three authors (AJ, CJI, and Charles Shey Wiysonge) had weekly meetings to discuss progress, findings, and next steps.

## 3. Results

### 3.1. Search results

**Figure 1:**
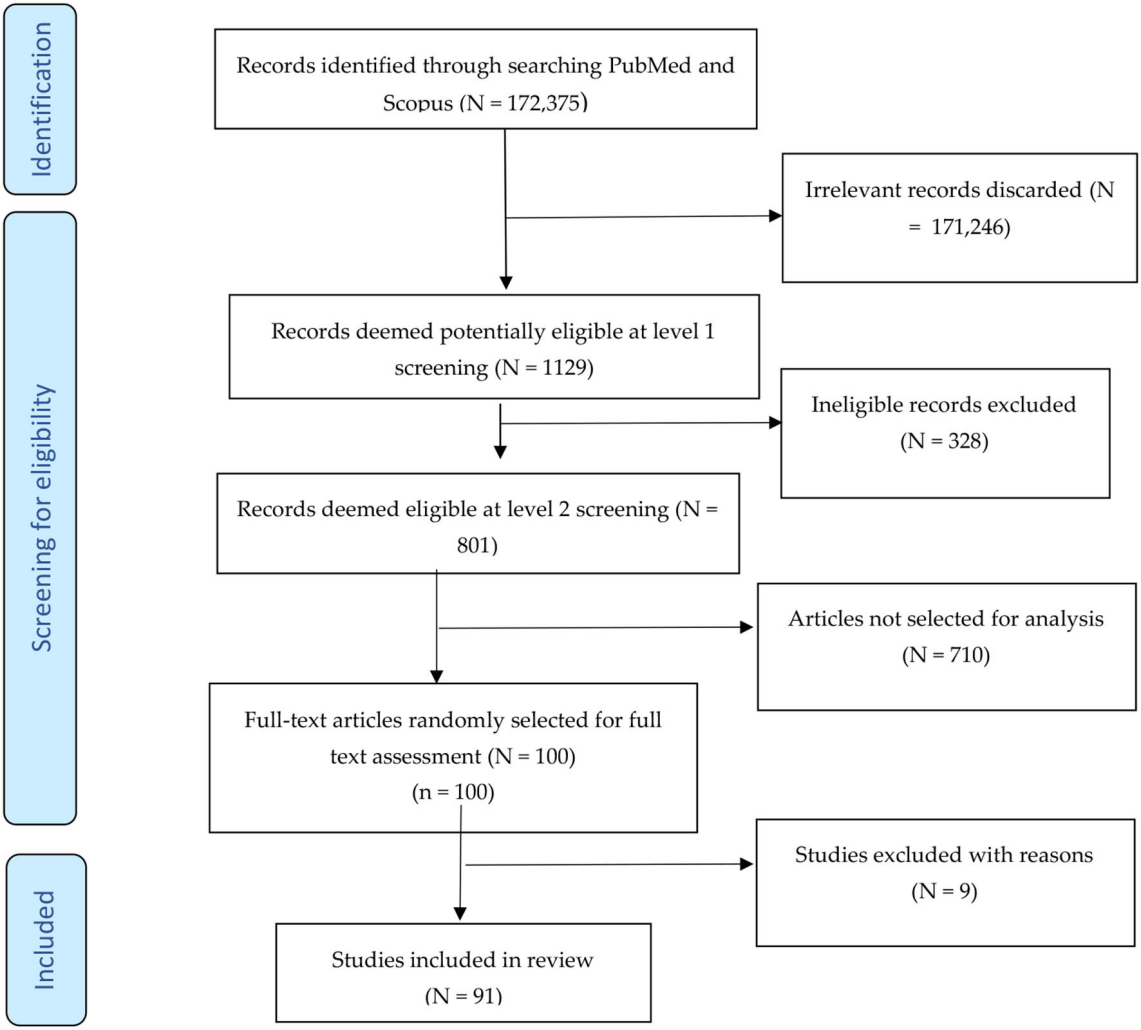
Study selection process for the scoping review.

Figure 1 shows the search and selection process for the scoping review. The literature search produced a total of 172,375 articles, including 11,695 from PubMed and 160,680 from Scopus. The first pair of researchers (AMN and ZPZ) screened these records and considered 1,129 publications to be potentially eligible for inclusion in the scoping review. The second pair (AJ and CJI) reviewed the 1,129 abstracts and found 801 to be potentially eligible for the review. Of the 801 potentially eligible articles, we randomly selected 100 publications whose full texts we obtained and assessed for eligibility. During the random selection, we used a systematic approach where we counted from the first article that appeared on Mendeley and selected every seventh publication. A systematic review involving all 801 potentially eligible studies is currently being undertaken. Of the 100 potentially eligible studies selected for assessment, 91 met inclusion criteria. Figure 1 shows the search and selection process.

### 3.2. WHO Regions

The characteristics of included studies are reported in detail in Supplementary Table 1.

Each of the six WHO regions had at least one publication included in the scoping review. Most of the included studies were conducted in WHO Region of the Americas (AMR) and European Region (EUR). The AMR had 28 studies (30.8%), which were conducted in the United States of America (Arizona, Florida, New York, Utah, Maryland, and Massachusetts), Mexico, Brazil, Chile, and Canada (19–46). EUR had 28 studies (30.8%) from France, the United Kingdom, Switzerland, Spain, Sweden, Turkey, Italy, and Germany (47–73). The Eastern Mediterranean Region (EMR) represented by Saudi Arabia, Iran, Iraq, and Pakistan had 14 studies (15.4%) (74–87). The South-East Asian Region (SEAR), represented by India, had 4 publications (4.3%) (88–91). The Western Pacific Region (WPR) represented by Australia, China and South Korea, had 11 publications (12.1%) (92–102). There was only one publication from the African Region (AFR), from Nigeria (1.1%) (103). Two publications included two countries each; one had China in WPR and UK in EUR and WPRO (2.2%) (104), while another involved Brazil in AMR and India in SEAR (105). Two of the publications (2.2%) were global studies and one study was conducted in Taiwan which does not fall under any WHO region (1.1%) (106–108).

### 3.3. Artificial intelligence methods and applications

#### 3.3.1. Single approaches

a. **Artificial intelligence:** Eight publications reported the AI broad technique in the use of developing therapy as the intervention for infectious diseases, for diagnosing COVID-19 and mental health conditions, and as screening tools for diabetes and cancer (19, 36, 57, 62, 75, 97, 104, 109). This technique was also used in designing vaccination, measuring and increasing medication adherence in non-communicable diseases, identifying genomic sequences, and developing drugs and vaccines for COVID-19 (19, 36, 57, 62, 75, 97, 104, 109).
b. **Machine learning:** Machine learning (ML) was reported in 46 studies to analyse, classify, diagnose, manage, monitor, and predict different health conditions or diseases (e.g., frailty, back pain, ischemic stroke, cancer, COVID-19, tuberculosis, diabetes, mortality, hypertension, mental health conditions, bacterial vaginosis, and heart disease) (21, 22, 25–27, 30, 33, 37, 39, 41, 43, 46, 47, 50, 52, 54, 56, 58, 60, 63, 65, 67, 70, 71, 73, 76, 77, 81, 82, 84, 87, 92, 96, 101, 103, 105, 108, 110). This approach was also used to create patient re-admission files, pre-authorization in health insurance, and for finding missed cases of disease; these all form a significant part in delivering medical care services (30, 76, 111).
c. **Deep learning:** Fifteen studies reported the use of deep learning (DL) in detecting cardiovascular disease, predicting mortality and cancer, diagnosing asthma, classifying cancer subtypes, pre-screening for COVID-19, and analyzing diseases like macular oedema (20, 24, 44, 55, 61, 64, 79, 83, 91, 93, 94, 98, 99, 106).
d. **Convolutional neural network:** Only one study mentioned the use of convolutional neural network (CNN) to diagnose cardiac diseases (112).
e. **Artificial intelligence optical microscopic -based technology:** One study reported the use of artificial intelligence optical microscopic (AIOM)-based technology in reproductive health to quantitatively measure sperm concentration and motility as well as seminal pH (107).
f. **Artificial neural network:** Two studies investigated the use of artificial neural network (ANN) to predict infectious disease (COVID-19) and non-infectious disease (hearing loss) among noise-exposed workers (72, 78).
g. **Bayesian network:** The Bayesian network (BN) is defined as a graphical tool that can be employed to build models from data and or expert opinion. This method can be used to predict, detect, and diagnose disease (113). In one study, this method was used in predicting the prognosis of suicidal behavior (23).
h. **Deep neural network:** The deep neural network (DNN) was used in two studies to predict the mortality of patients in palliative care and classify breast cancer (29).
i. **Fuzzy K-means clustering algorithm:** The Fuzzy K-means clustering algorithm (FKCA) was used in one study to detect and classify cataract in normal, cataract, and post-cataract optical images (90).
j. **COVID Inception-ResNet model deep learning:** Almalki 2021 explored COVID Inception-ResNet (CoVIR-Net) model deep learning as a method for using chest X-rays to diagnose COVID-19 (40).

#### 3.3.2. Combined approaches

Combining deep learning and machine learning with other approaches: Some of the included studies investigated the use of a combined AI approach for delivering medical care services. Three of those studies used DL and ML to diagnose and predict COVID-19, cardiovascular disease risk, hazardous drinkers, and the severity of alcohol-related problems (49, 102, 114). ML was also used in combination with artificial neural network to diagnose, predict and manage prognosis of nervous system disorders (69). One study reported the use of DL and NLP to quantify left and right ventricular dysfunction from electrocardiograms. DL was also used together with neural network mode to diagnose COVID-19 in chest X-ray images (68). In another study, DL was used with multi-head attention (MHA), Long Short-Term memory (LSM), and CNN (45).

### 3.4. Health conditions assessed using AI applications

A total of 21 studies focused on infectious diseases of various types (33, 40, 42, 45, 49, 52, 64, 65, 68, 72, 75, 80, 82, 83, 91, 100, 104, 106, 108, 114). Eighteen of these studies focused on COVID-19 while the remaining ones were on tuberculosis. Thirteen studies targeted cardiovascular diseases (including stroke, hypertension, ventricular dysfunction, and heart function) (31, 34, 39, 44, 46, 51, 59, 61, 71, 76, 87, 95, 112). These were mostly experimental studies conducted in WHO AMR, EMR, EUR, SEAR, and WPR regions for prediction and diagnostic purposes. Six studies focused on cancers, including prostate, lung, skin, and breast (48, 67, 73, 79, 92, 115). They were conducted in AFR, EUR, SEAR, and WPR. These investigations were mostly experimental studies for prediction and diagnostic purposes. There were 21 studies on conditions on assorted conditions ( (22, 24, 26, 27, 30, 35, 36, 39, 41, 54, 55, 58, 60, 66, 69, 70, 78, 81, 85, 86, 98). These conditions include injuries, diet, sepsis, and drug overdose. Eight studies were on mental and cognitive health problems including various forms of depression and dementia (37, 50, 53, 57, 84, 101). These studies were conducted in EUR, AMR, and SEAR. The studies mostly focused on prediction and diagnosis. Only one study was on reproductive health where AI was used for diagnostic purposes in men (107).

## 4. Discussion

This paper highlights currently used AI techniques and applications. The AI techniques identified include machine learning, deep learning, convolutional neural network, artificial intelligence optical microscopic-based technology, artificial neural network, Bayesian network, deep neural network, Fuzzy K-means clustering algorithm, COVID Inception-ResNet model deep learning, natural language processing, neural network mode, and long short-term memory. The AI techniques were used for four main groups of medical care services, including: (i) detection and diagnosis; (ii) classification; (iii) treatment, support, and prognosis; and (iv) management of research and clinical care. Most of the studies focused on the use of machine learning and distance learning as applications to detect and diagnose different diseases. These include infectious diseases (COVID-19 and tuberculosis); cardiovascular diseases (ischemic stroke, cardiomyopathy, hypertension); metabolic diseases (diabetes); cancers (breast, prostate, diffuse gliomas, skin); and mental diseases (schizophrenia, dementia, suicidal behavior). These techniques have also been used in hospital and research administration. Generally, machine learning and deep learning show the possibility of being used to improve the competence of clinical and research procedures which will be beneficial to good health.

It is important to note that these investigations were predominantly conducted in the WHO Region of the Americas and the European Region than in other regions. We only found one study conducted in Africa in this sample of studies. The difference concerning distributions of publications in the WHO regions showed that there is a lack of research conducted in low and middle-income countries around this field of study. Most healthcare facilities with lack of resources infrastructure, specifically, low-and middle-income countries (LMICs), do not have digital infrastructure to implement AI in their settings. HICs on the other hand, with highly skilled healthcare workers who can explain AI results regarding clinical scenario while in LMICs, all this may be lacking. It is important to note that for AI to be fully functional, it first must be available, accessible, and sustainable (10). A good example is where AI is applied in radiology, where its functionality involves services like imaging hardware, servers, information technology, quick internet service, picture storage and communication system, electronic medical records, and cloud services. This shows that it would be challenging to establish AI in low resource settings where is lack of experts to interpret its outputs and to apply them appropriately (10). Therefore, if AI would be implemented successfully in LMICs, healthcare professionals would need to be educated and trained on how to use the technology. AI implementation involves a lot of processes for it to perform well and important aspect is using the same data from the same source as the training set. Currently, most data to develop AI come from HICs, with some from middle-income countries (116). Health experts recommend that LMICs regulate AI processes together with global health organizations who would give them support (116). This would help healthcare workers in LMICs with successfully implementing and applying this technology. Other benefits of applying AI in healthcare in LMICs would be improving the state of health systems and decreasing medical costs such as those of screening (117). Furthermore, costs related to treatment plans that need expensive tools and specialized expertise especially in rural and remote settings (117).

There is, however, a relatively high number of publications in the Eastern Mediterranean Region, mainly involving studies conducted in Saudi Arabia. The lack of studies from under resourced regions may also be due to insufficient resources and AI knowledge among healthcare practitioners, especially in the WHO African Region. In view of the above, AI can be greatly beneficial to healthcare services in LMICs although its introduction would be quite different from what is done in HICs (10). For this scoping review, we used a random sample of currently available peer-reviewed publications to map out and synthesize the available evidence on the use of AI to deliver medical care services globally and regionally. The review suggests that AI is predominantly applied in high-income countries, with its use still emerging in low-resource settings (such as the WHO African Region) perhaps because health institutions in these settings do not have the infrastructure for use of this technology. This scoping review suggests that there is value in undertaking a systematic review and will proceed with conducting the review on the topic. The systematic review will focus on discussing the use and effectiveness of AI in delivering healthcare services. Furthermore, the review will discuss and compare applications including diagnosis and treatment of disease, patient engagement and adherence, and administrative services.

Linear models, including linear regression, multiple regression and multivariate linear regression models have also been used in medical research. Linear and multiple regression models are methods used to predict and assess interactions between the different datasets (118). The linear regression model is also used to address different research questions and study aims (118). Multiple linear regression has been used to predict the length of stay for patients undergoing treatment heart disease, diabetes, hypertension, cancer, and laparoscopic appendectomy by (119). Multivariate Regression Analysis of Variance) is a multiple test that combines all the tests on the significance of the single regression coefficients (120).

## 5. Strengths and limitations of the study

The present review achieved its aim of mapping out and synthesizing literature on the use of AI methods in medical care. However, it is important to note that a broader review, i.e., a systematic review, would further illuminate the gaps in literature. A limitation of this study is that it did not evaluate the effectiveness of the different AI techniques in medica care services. A research question on the effectiveness of AI techniques within the different health issues require that another review be conducted. In review of the above, it must be considered that conducting rapid rather than systematic reviews to address effectiveness questions would be beneficial since there are constant publications around this field. A rapid review would ensure that relevant evidence is collected and disseminated in time.

## 6. Conclusions

Currently available evidence shows that the AI techniques are commonly used to deliver medical care services, especially in HICs. The commonly used methods for the detection, diagnosis, management, and monitoring the prognosis of different diseases are deep learning, and machine learning. The use of AI in the various health issues, namely, infectious diseases (COVID-19 and TB); metabolic diseases (diabetes); cardiovascular disease (ischemic stroke, cardiomyopathy, hypertension), cancers (breast, prostate, diffuse gliomas, skin) and mental diseases (schizophrenia, dementia, suicidal behavior) has shown positive outcomes. Other conditions in which the application of AI has shown positivity, include, frailty, low back pain, oral leucoplakia, open wound mortality, pressure injuries, primary progressive aphasia, dementia, lung function, asthma, and growth hormone deficiency. Further research is required in the use of other AI techniques to advance medical care delivery, especially in WHO African, Eastern Mediterranean, South-East Asian, and Western Pacific regions. AI methods are becoming more available for researchers and clinicians to apply, and it is probable that this field will continue to grow.

## Author contributions

AJ designed the search strategy with an important input from CI-J and CW. AJ and CI-J conducted literature searches. AN and ZZ screened the search output and AJ and CI-J re-screened the articles. AN, ZZ, AJ, CI-J, and EM extracted data from eligible articles. AJ, CI-J, and JO wrote the first draft of the manuscript. CW, JO, DM, LM, NI-A, and HB guided the project and critically revised the intellectual content of the manuscript. All authors have read and agreed to the published version of the manuscript.
